# Syndrome de Melkersson-rosenthal: une entité rare à ne pas méconnaitre

**DOI:** 10.11604/pamj.2015.21.33.6962

**Published:** 2015-05-18

**Authors:** Rim Klii, Wafa Chebbi

**Affiliations:** 1 Service de Médecine Interne et d'Endocrinologie, CHU Fattouma Bourguiba Monastir, 5000 Monastir, Tunisie; 2 Service de Médecine Interne, CHU Taher Sfar Mahdia, 5100 Mahdia, Tunisie

**Keywords:** Syndrome de Melkersson-Rosenthal, œdème oro-facial, paralysie faciale, Melkersson-Rosenthal syndrome, orofacial edema, facial paralysis

## Image en medicine

Le syndrome de Melkersson-Rosenthal est une entité clinique rare, définie par la triade oedème oro-facial, paralysie faciale récurrente et langue plicaturée. Cette triade peut être incomplète ou apparaître de manière différée dans le temps. Le diagnostic est confirmé par l'histologie. Il s'agit d'une entité complexe dont la prise en charge est difficile en raison de son polymorphisme clinique et de l'absence d’étiopathogénie identifiée. Nous rapportons une observation rare de syndrome de Melkersson-Rosenthal dans sa forme complète. Il s'agissait d'une patiente âgée de 30 ans, sans antécédents pathologiques notables, qui consultait pour un oedème oro-facial récidivant. L'examen révélait un oedème facial, indolent, élastique et chaud, une macrochélite avec une paralysie faciale droite. L'examen de la cavité buccale montrait une langue plicaturée avec des fissurations transversales. Devant l'association de paralysie faciale, oedème oro-facial et langue fissurée, le diagnostic de syndrome de Melkersson-Rosenthal dans sa forme complète était retenu. La biopsie labiale a confirmé le diagnostic en montrant: un chorion avec des nodules riches en lymphocytes bordés d'une couronne plasmocytaire et histiocytaire sans nécrose caséeuse dans un tissu conjonctif oedémateux et fibrosé par endroit. Une corticothérapie par voie générale de courte durée, associée à des injections intra-lésionnelles de Triamcinolone (Kénacort), était instaurée aboutissant à la sédation des oedèmes.

**Figure 1:**
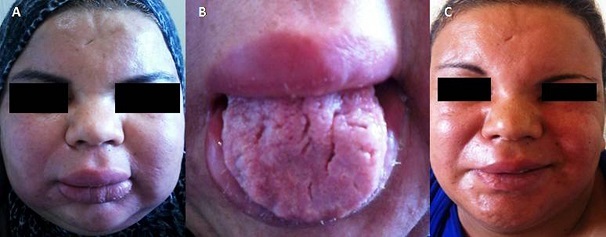
(A) œdème oro-facial; (B) langue plicaturée; (C) paralysie faciale droite

